# Brain toxoplasmosis after HLA haploidentical transplantation for acute myeloid leukemia

**DOI:** 10.1002/jha2.454

**Published:** 2022-05-16

**Authors:** Takafumi Tsushima, Rikako Tabata, Kentaro Narita, Koichi Honma, Kengo Takeuchi, Kosei Matsue

**Affiliations:** ^1^ Division of Hematology/Oncology Kameda Medical Center Chiba Japan; ^2^ Department of Clinical Pathology Kameda Medical Center Chiba Japan; ^3^ Division of Pathology The Cancer Institute, Japanese Foundation for Cancer Research Tokyo Japan; ^4^ Department of Pathology The Cancer Institute Hospital, Japanese Foundation for Cancer Research Tokyo Japan

1

A 66‐year‐old man with acute myeloid leukemia with a complex karyotype achieved complete remission with idarubicin + cytarabine (Arac) (3 + 7) followed by high‐dose Arac for three cycles which were followed by haploidentical stem‐cell transplantation with fludarabine, intravenous busulfan, and low‐dose total body irradiation. Post‐transplant cyclophosphamide was used for the prevention of graft‐versus‐host disease (GVHD). Neutrophil engraftment was observed on day 21. On day 32, the patient developed acute gastrointestinal GVHD and was started on prednisolone (1 mg/kg). GVHD gradually improved, and prednisolone was reduced, but on day 72, the patient developed pulmonary GVHD and was treated with a high‐dose corticosteroid. Around the same time, he began to experience disorientation, which worsened rapidly. His respiratory condition gradually improved with immunosuppressive drugs and artificial respiration, but his disorientation persisted. Computed tomography (CT) of the brain showed no abnormalities, and magnetic resonance imaging showed multiple high‐signal findings with myelography. Examination of the cerebrospinal fluid was negative for both bacteria and viruses. Because serum toxoplasma immunoglobulin M (IgM) was elevated, that is 1.4 IU/ml (range: 0.0–0.8 IU/ml), we considered the possibility of cerebral toxoplasmosis and administered sulfamethoxazole/trimethoprim; however, there was no improvement in consciousness, and the patient died on day 99. A brain CT scan taken after his death showed multiple small calcified lesions in his brain (Figure 1A), and autopsy findings showed infarct‐like necrosis (Figure 1B) and *Toxoplasma* cysts (bradyzoites) in the bilateral basal ganglia, cerebral cortex, and subcortical areas (Figure 1C). Immunostaining by anti‐*Toxoplasma gondii* antibody was positive (Figure 1D). Multiple cerebral calcified lesions are a typical picture of congenital cerebral toxoplasmosis, but they are rare in cases of acquired cerebral toxoplasmosis in immunosuppressed adults. Moreover, calcification in a short period, as in this patient, has rarely been reported before.

**FIGURE 1:**
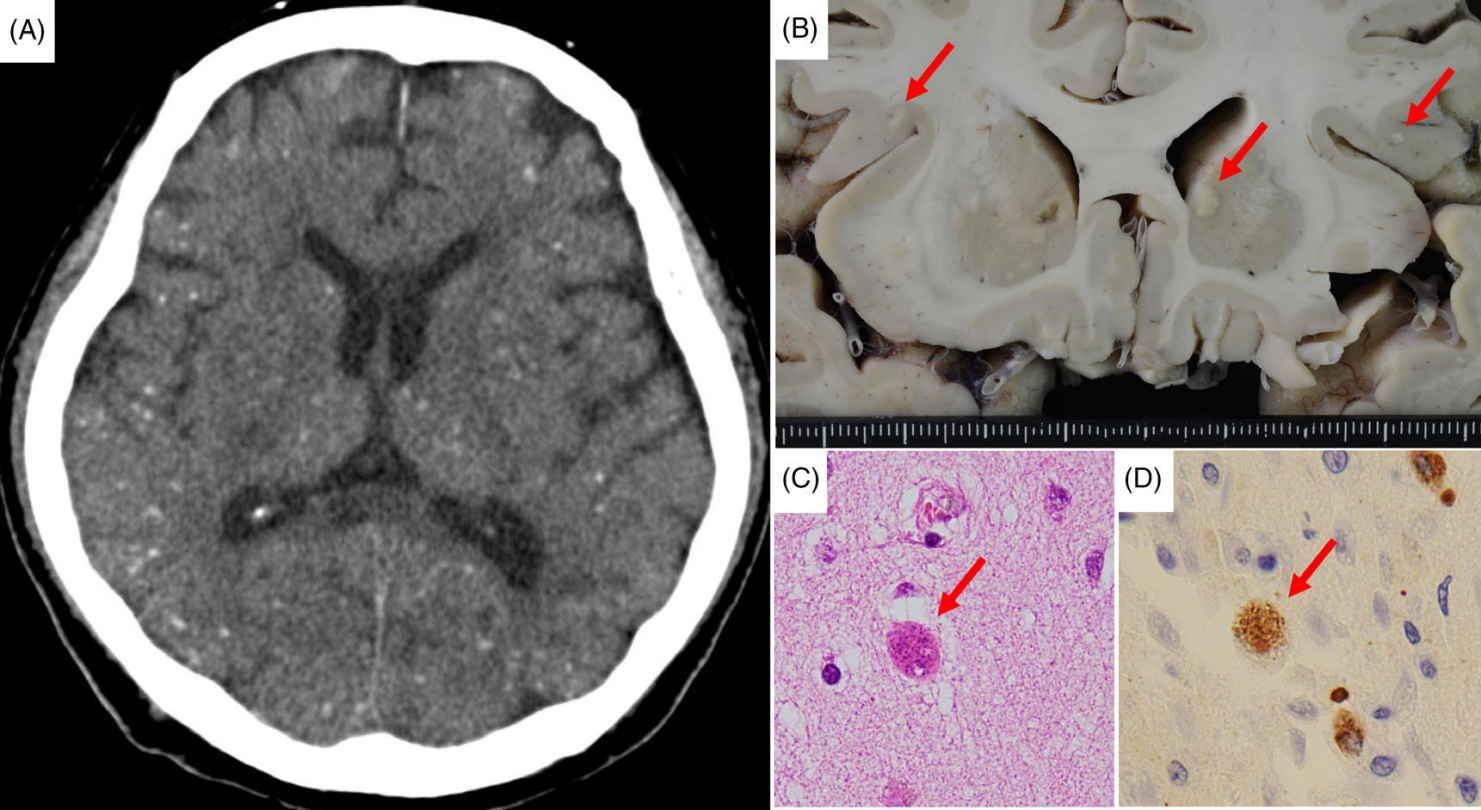
Brain toxoplasmosis after human leukocyte antigen (HLA) haploidentical transplantation for acute myeloid leukemia. (A) A brain computed tomography (CT) scan taken after his death showed multiple small calcified lesions in his brain. (B) Autopsy findings showed infarct‐like necrosis. (C) *Toxoplasma* cysts (bradyzoites): hematoxylin and eosin (H&E) stain, 400×. (D) *Toxoplasma* cysts: anti‐*Toxoplasma gondii* antibody stain, 400×

## FUNDING

The authors received no specific funding for this work.

## CONFLICT OF INTEREST

The authors declare they have no conflicts of interest.

## ETHICS STATEMENT

Ethical approval was not sought for the present study because this study is only a single case report with complete removal of all identifying information. The patient provided consent for the publication of this case with the removal of all identifying information to remain anonymous and retain his privacy.

## AUTHOR CONTRIBUTIONS

Takafumi Tsushima designed the study, collected data, wrote the manuscript, and provided patient care. Rikako Tabata and Kentaro Narita provided patient care. Koichi Honma and Kengo Takeuchi performed pathology. Kosei Matsue supervised this paper. All authors reviewed and approved the manuscript.

